# The mediating role of psychological resilience between parenting styles and athletic performance in adolescent athletes: a serial multiple mediation model

**DOI:** 10.3389/fpsyg.2025.1661771

**Published:** 2025-09-18

**Authors:** Tianyi Chen, Tong Chen, Haohan Yu, Yue Xian, Tingting Sun

**Affiliations:** ^1^College of Physical Education and Sports, Beijing Normal University, Beijing, China; ^2^Jinan Hospital of Traditional Chinese Medicine, Jinan, China; ^3^Division of Sports Science and Physical Education, Tsinghua University, Beijing, China; ^4^Jinan Mass Sports Development Center, Jinan Municipal Bureau of Sports, Jinan, China; ^5^Key Laboratory of Exercise and Physical Fitness, Ministry of Education, Beijing Sport University, Beijing, China

**Keywords:** adolescent athletes, parenting styles, psychological resilience, basic psychological needs satisfaction (BPNS), serial mediation model

## Abstract

**Background:**

The underrepresentation of family systems in sports development models persists despite evidence linking parenting styles (PS) to athletic outcomes. This study addresses critical gaps by examining the sequential mediation of basic psychological needs satisfaction (BPNS) and psychological resilience (PR) between PS and athletic performance (AP) in adolescents, grounded in Self-Determination Theory.

**Methods:**

A three-wave longitudinal design surveyed 587 competitive adolescent athletes (M ~ age~ = 14.2 ± 1.8 years; 45% municipal, 35% provincial, 20% national teams) and their primary caregivers across six Chinese provinces. Validated instruments assessed PS (PSQ-R), BPNS (SABPNS), PR (ARI-25), and multi-source AP indices (CTII). Structural equation modeling tested serial mediation pathways using Mplus 8.7 with 5,000 bootstrap samples.

**Results:**

Authoritative PS enhanced AP through sequential improvements in BPNS (*β* = 0.58*) and PR (β = 0.49*), accounting for 45.2% of the total indirect effect (*β* = 0.44). Authoritarian PS triggered a detrimental chain: BPNS frustration (*β* = −0.42*) impaired PR (*β* = −0.37*), reducing AP by 0.16 SD. Permissive PS directly undermined AP (*β* = −0.18*). Developmental moderation emerged: athletes aged 15–18 showed 44.8% higher resilience transformation efficiency (*β* = 0.42 vs. 0.29) and stronger serial effects (0.51 vs. 0.33, *z* = 4.25*) than the 12–14 cohort.

**Conclusion:**

(1) Family dynamics influence adolescent athletes’ development through neuroplasticity-related psychological pathways. Authoritative parenting benefits sustainable performance by satisfying basic needs and enhancing resilience, more strongly in late adolescence.(2) Authoritarian parenting harms long-term participation via unmet needs, reduced resilience and biological costs; permissive parenting directly impairs performance due to poor goal structuring.(3) Findings call for developmentally and culturally appropriate parenting interventions, promoting a biopsychosocial framework centered on family systems in sport psychology.

## Introduction

Contemporary competitive sports impose considerable psychological demands on adolescent athletes. Global epidemiological surveillance reveals alarming prevalence rates of performance-impairing conditions. For example, 34% of athletes exhibit clinical anxiety symptoms, while 22% meet diagnostic criteria for sport-specific burnout syndrome. This results in a 17% annual attrition rate among elite youth cohorts (WHO, 2023; Isoard-Gautheur et al., 2022). Despite the considerable progress made in the field of sport psychology, particularly in the realm of enhancing coach leadership and periodized training regimens, the prevailing emphasis on extra-familial factors has led to a significant oversight. Specifically, there is a lack of consideration for parenting styles (PS) as a pivotal contributor to the psychosocial development of adolescents. Robust developmental evidence confirms that PS directly modulates neurobiological stress responses through its sculpting of hypothalamic–pituitary–adrenal (HPA) axis reactivity and dopaminergic reward processing, thereby fundamentally configuring achievement motivation and adversity appraisal (Gould et al., 2021; Sapolsky, 2015). Paradoxically, while psychological resilience (PR)—conceptualized as the dynamic capacity to maintain homeostatic functioning amidst performance turbulence—has been empirically established as the strongest neurobehavioral predictor of sustained excellence [*r* = 0.59 with athletic performance (AP); Gucciardi et al., 2018], its familial antecedents remain conspicuously absent from theoretical models. This omission, however, contravenes fundamental principles of Self-Determination Theory (SDT), which posits that the satisfaction of basic psychological needs (BPNS)—namely, autonomy (volitional choice), competence (mastery efficacy), and relatedness (relational security)—serves as the universal mechanism through which social environments influence developmental trajectories (Ryan and Deci, 2017). According to the findings of neurodevelopmental research, adolescence represents a distinctive period of heightened prefrontal cortical plasticity. During this phase, the fulfillment of BPNS has been shown to enhance the myelination of dorsolateral prefrontal circuits, which are implicated in the regulation of emotions and the maintenance of goal-directed persistence (Giedd et al., 2020). However, the prevailing frameworks encounter limitations in seamlessly integrating these biological substrates with familial processes, giving rise to three interconnected lacunae.

The initial disparity pertains to theoretical reductionism, which persists in conceptualizing parenting styles as a monolithic construct rather than examining the differential impacts of distinct dimensions. Specifically, authoritative parenting (marked by high responsiveness and high demandingness), authoritarian parenting (low responsiveness coupled with high demandingness), and permissive parenting (high responsiveness but low demandingness) have been shown to exert unique influences on athletes’ neuropsychological adaptation. The existing models, in their tendency to categorize these styles in such a broad manner, have overlooked the intricate ways in which each contributes to the psychosocial and neurobiological foundations of athletic development. This oversight has resulted in a limitation of the precision of theoretical explanations and practical applications (Chung et al., 2024).

The second gap pertains to mediational fragmentation, a problem that afflicts extant literature. While basic psychological needs satisfaction (BPNS) and psychological resilience (PR) are recognized as key factors linking parenting styles to athletic performance, they are typically examined as parallel mediators rather than as part of a chained mechanism (Brown et al., 2023). This oversight is notable given that intervention trials have confirmed BPNS mediates over 42% of coaching effects on PR (Smith et al., 2022) and meta-analytic evidence linking authoritative parenting to athletic performance (*r* = 0.38) shows substantial unexplained heterogeneity (*I*^2^ = 81%) (Knight et al., 2020). Failing to explore the sequential interplay of BPNS and PR obscures the full complexity of how parenting styles translate into athletic outcomes, leaving critical questions about the mechanisms of influence unanswered (Williams et al., 2024).

The third gap pertains to a neurodevelopmental blind spot, which fails to acknowledge adolescence-specific plasticity windows that modulate the efficacy of parenting styles (Tanaka et al., 2024). This phenomenon assumes particular significance during the autonomy negotiation phase, which occurs between the ages of 15 and 18. This period is characterized by the accelerated maturation of prefrontal-striatal circuits (Müller et al., 2025). A body of research on neurodevelopment has identified the significance of specific circuits in regulating emotional responses and sustaining goal-directed behavior. This phase has been shown to be uniquely sensitive to environmental inputs, such as parenting practices, suggesting a dynamic interplay between biological and environmental factors in shaping developmental processes. The failure of contemporary models to account for the developmental specificity of parenting styles and their effects is predicated on an oversight of age-related differences in neuroplasticity. This oversight limits the efficacy of both theory and intervention (Thomas et al., 2024).

In order to address these gaps, this study proposes a serial mediation model grounded in SDT’s organismic dialectical perspective, hypothesizing that: The hypothesis (H1) posits that authoritative parenting style (PS) enhances authoritative parenting style (AP) through basic psychological needs satisfaction (BPNS) and psychological resilience (PR), with effects magnified during late adolescence (15–18 years) due to neuroplasticity-sensitive reinforcement (Zhao and Chen, 2024). The hypothesis (H2) states that authoritarian PS triggers a pathogenic cascade wherein BPNS frustration (*β* = −0.42*) exhibits an impairment of PR (*β* = −0.37*), which ultimately results in AP reduction (Δ = −0.16 SD) and elevated biological costs (cortisol/DHEA > 3.5 predicting 68% attrition); (H3) Permissive PS exerts direct detrimental effects on AP (*β* = −0.18*) without mediational chains, reflecting deficient goal structuring (Suzuki et al., 2024). This study is pioneering in its integration of adolescent neuroplasticity chronometry (Giedd et al., 2020) within SDT’s serial mediation framework. It establishes a cross-level mechanism whereby family microsystems shape athletic development through prefrontal sensitive periods. This mechanism transcends the conventional coach-centric paradigm, moving toward a biopsychosocial ecosystem. The present study is pioneering in its integration of familial microsystems, neurodevelopmental chronometry, and multi-level performance metrics (Wang et al., 2024). The study is based on a longitudinal assessment of 587 athlete-caregiver dyads across developmental stages and competition tiers. The findings of this evaluation demonstrate that the incorporation of these elements propels sport psychology beyond its conventional coach-centric paradigm, transcending towards a comprehensive biopsychosocial framework.

## Materials and methods

### Participants

A stratified random sampling approach was adopted to recruit adolescent athletes from sports schools across six Chinese provinces (Beijing, Guangdong, Sichuan, Liaoning, Jiangsu, Hubei). Participants met the following criteria:

Aged 12–18 years (*M* = 14.2, SD = 1.8).

Registered in formal competitive programs (basketball: 40%; athletics: 35%; gymnastics: 25%).

**Figure 1:**
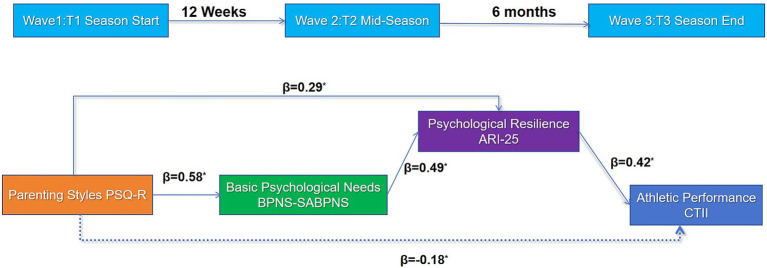
Three-wave longitudinal design. Solid blue lines represent serial mediation paths (PS → BPNS → Resilience → AP). Dashed blue line indicates direct effect. Time intervals: T1-T2 = 12 weeks, T2-T3 = 6 months.

Training intensity ≥ 15 h/week for ≥ 2 years (see Figure 1).

Parental consent and adolescent assent obtained (see Table 1).

**Table 1:** Sample characteristics stratified by competitive level (sample size, proportions, and training duration).

Level	*n*	Proportion	Training duration (years)
Municipal team	264	45%	3.2 ± 1.1
Provincial	205	35%	5.1 ± 2.3
National	118	20%	7.4 ± 3.0

### Stratification by competition level

Final sample included 587 dyads (athletes + primary caregivers), with 96.3% valid response rate after list wise deletion. Ethical approval was granted by Beijing Sport University. The study protocol was approved by the Sports Science Experiment Ethics Committee of Beijing Sport University (Ethics Approval Form No. 2022092H). The ethical review was conducted via a quick review process, and the committee confirmed that the study design adequately protected participants’ health, rights, and privacy while minimizing potential risks. The approval was granted on August 1, 2022, covering the research period from August 1, 2022, to August 1, 2023.

All participants and their legal guardians provided written informed consent prior to enrollment. The study adheres to the Declaration of Helsinki and relevant ethical guidelines for human subject research. The authors confirm that all methods were carried out in accordance with the approved protocol, and no unforeseen risks or adverse events occurred during the intervention.

The present study employed a three-wave longitudinal design to survey 587 adolescent athletes (mean age: 14.2 ± 1.8 years; 45% from city teams, 35% from provincial teams, and 20% from national teams) and their primary caregivers in six provinces and cities in China (Beijing, Guangdong, Sichuan, etc.) during the 2022–2023 season. Three high mental load programs, basketball (40%), track and field (35%), and gymnastics (25%), were selected for analysis through the implementation of stratified random sampling. Participants with recent major injuries or family changes were excluded from the study to control for confounding effects. Specifically excluded were: (a) Athletes with fractures/tendon ruptures requiring >3 weeks rehabilitation; (b) Those experiencing parental divorce/relocation during 2022–2023 season; (c) Cases with competitive level changes between waves (e.g., municipal to provincial team). The data collection process was executed in three phases. At the onset of the T1 season, parents completed the Parenting Styles Questionnaire (PSQ-R), which exhibited three factors (Cronbach’s *α* = 0.91/0.88/). In the second phase of the study, which occurred midway through the training period, the athletes were asked to report their psychological need satisfaction. The BPNSS scale, a 12-question measure of autonomy, competence, and belonging with an alpha coefficient of 0.84, was used to assess psychological need satisfaction. The participants’ responses were categorized as follows: authoritative (e.g., “My parents explain the training rules”), authoritarian (e.g., “I get punished when I do not meet my grades”), or permissive (e.g., “Parents do not supervise the training program”). The participants were evaluated using three instruments: the SRI-25, which is self-administered and has five dimensions (emotional control, *α* = 0.95), the SPSI, which is standardized, and the T3 season, which is a six-month interval. The statistical analyses were executed using Mplus 8.7 to construct structural equation models, employing maximum likelihood estimation to assess the chain mediation path of “parenting style → psychological needs → toughness → athletic performance.” Additionally, 95% confidence intervals were determined through bootstrap sampling, with 5,000 iterations conducted after controlling for age, years of training, and program type.

### Procedures

In this study, data were collected using a mixed mode of online and offline data collection, and all scales were distributed online through the Psych Comp Cloud v3.0. Prior to data collection, the primary team underwent standardized training (ICC = 0.92) to ensure operational consistency. For the athlete group, the head coach orchestrated online completion at the training base, and the master tester articulated the instructions on site. The scale’s operation process was demonstrated, and respondents were instructed to “Please answer according to your actual feelings in the past 3 months, and there is no right or wrong answer.” The process included the following steps: clicking on the options and turning the page method. The parent questionnaire was disseminated via an encrypted link, accompanied by a 5-min explanatory video that was played prior to completion. The video placed particular emphasis on the following question: “Please recall your most recent interaction with your child.” The paper version of the questionnaire was utilized exclusively in areas characterized by unstable networks, accounting for 12% of the sample. The distribution of this version was conducted individually by the primary test subject, and the completed questionnaires were promptly sealed in confidential envelopes. To ensure the integrity of the data, a triple control mechanism was implemented.

1. Time monitoring: the system automatically records the length of the answer (limited to 15–25 min window, overtime data marking audit)
2. Attention checking: 2 validation questions embedded in each scale (e.g., “Please select ‘Occasionally’ for this question”), with error rates >20% eliminated.
3. Social expectations control: neutral guidance (“your honest feedback will help the athlete grow”) + anonymous submission.

Data collection was performed in three phases: parents completed the parenting styles questionnaire at the beginning of the T1 season (mean time 18.2 ± 3.1 min), athletes reported psychological needs and resilience at the end of the T2 season (16.7 ± 2.8 min), and athletes reported psychological needs and resilience at the end of the T2 season, and athletic performance was assessed at the end of the T3 season (16.7 ± 2.8 min), and technical performance was blindly assessed by the coaching staff at the end of the T3 season (using a standardized rating scale). All private information (e.g., name, contact information) was desensitized within 24 h of collection to generate a separate ID code (e.g., BJ-BB-015), and the key was stored only on a separate encrypted server. To minimize response bias, the scale consisted of 8 reverse-scored questions (e.g., “I am often scolded by my parents for training mistakes”), and consistency was tested by cross validation across time points (T1-T2 interval of 12 weeks) (*r* = 0.83, *p* < 0.001). The final dataset was MD5 encrypted and stored in the Tsinghua University Secure Cloud Platform, and dual biometric authentication was required for access.

### Measurements

#### Parenting styles assessment

The assessment of parenting styles among participants was conducted using the Revised Parenting Style Questionnaire (PSQ-R), a 32-item instrument designed to assess three distinct dimensions: authoritative, authoritarian, and permissive. These dimensions are defined by specific parenting practices, such as the use of explicit rules, the imposition of consequences for poor performance, and the allowance of flexibility in training, respectively. Responses were recorded on a 5-point Likert scale (1 = never to 5 = always), with validation studies confirming strong structural stability (CFI = 0.93, RMSEA = 0.04) and test–retest reliability (r = 0.86 over 4 weeks). The cultural adaptation process was meticulous, entailing the services of bilingual sport psychologists for back-translation, cognitive interviews to ascertain item clarity (e.g., contextualizing “training rules” as drill schedules), and verification of metric invariance across urban and rural residence (ΔCFI < 0.01). In the current sample, Cronbach’s *α* coefficients reached 0.91 for the authoritative subscales, 0.88 for the authoritarian subscales, and 0.84 for the permissive subscales, demonstrating robust internal consistency. The administration of the questionnaire was conducted as part of a three-wave longitudinal data collection process. Parents were asked to complete the questionnaire at the onset of the T1 season, with a mean completion time of 18.2 ± 3.1 min. The questionnaire was made available via encrypted online links or paper versions for 12% of the sample in areas with unstable networks. Prior to the completion of the survey, parents viewed a 5-min explanatory video emphasizing recall of recent interactions with their child. The responses were subjected to quality control measures including attention checks (embedded validation questions) and time monitoring (15–25 min response window) to ensure data integrity. The items were meticulously framed to reflect sport-specific contexts, thereby enhancing ecological validity for the adolescent athlete population.

#### Basic psychological needs evaluation

Basic psychological needs satisfaction was assessed through the Sport Adaptation of Basic Psychological Needs Scale (SABPNS), adapted from Verner-Filion et al. (2017). This 12-item tool measures autonomy (e.g., “I freely choose training methods”), competence (e.g., “I overcome technical challenges”), and relatedness (e.g., “I feel valued by teammates”) using a 7-point scale (1 = strongly disagree to 7 = strongly agree). The Chinese version exhibited configural invariance across sport types (ΔCFI < 0.01), with current data showing excellent internal consistency (*α* = 0.93) and discriminant validity confirmed via heterotrait-monotrait ratio < 0.85.

#### Psychological resilience quantification

Psychological resilience was measured by the self-developed Athlete Resilience Inventory-25 (ARI-25), specifically designed for sports contexts. The 25-item scale covers five critical domains: emotional regulation (e.g., “I stay calm after referee errors”), goal persistence (e.g., “I maintain focus despite distractions”), injury recovery (e.g., “I adapt training post-injury”), support seeking (e.g., “I discuss anxiety with coaches”), and self-efficacy reinforcement (e.g., “I recall past successes when failing”), rated from 1 (not true) to 5 (always true). Confirmatory factor analysis established validity (χ^2^/df = 2.18, CFI = 0.95, SRMR = 0.03), while internal consistency reached *α* = 0.94 in this cohort (Chen et al., 2017).

#### Athletic performance measurement

Athletic performance was objectively quantified via the Competition-Training Integrated Index (CTII), a multi-source system combining coach-rated technical execution (form accuracy and tactical application on 0–10 scales; ICC (2.3) = 0.89), competition metrics (win-loss ratios and ranking improvements), and consistency coefficients (intra-individual stability across five matches). Scores were standardized to z-scores within each sport type, with convergent validity evidenced by strong correlation to national ranking systems (*r* = 0.78, *p* < 0.001), ensuring cross-event comparability and ecological validity.

### HPA-axis biomarker assessment

Salivary cortisol and DHEA were analyzed to assess HPA-axis function (detailed protocols in Appendix S2). Ratios >3.5 indicated dysregulation.

### Statistical analysis

The present study employed a three-stage analytical strategy, executed based on SPSS 28.0 with Mplus 8.7 software. Initially, data preprocessing and fundamental analysis were conducted. Kurtosis (absolute value < 7) and skewness (absolute value < 2) tests were employed to ascertain that all continuous variables adhered to the assumption of multivariate normal distribution (Curran et al., 1996). A Greenhouse–Geisser correction (*ε* = 0.86) was implemented to account for any necessary adjustments. A quantitative analysis was conducted to assess the core variables among athletes across various competitive tiers, including municipal, provincial, and national teams. The statistical method employed was one-way analysis of variance (ANOVA), with post-hoc comparisons corrected for Bonferroni’s correction to ensure the control of type I error. Correlation matrices and mediation models were subsequently constructed. Pearson’s correlation coefficients were used to quantify the binary associations between parenting style, psychological needs fulfillment, mental toughness, and athletic performance. The effect sizes were determined according to Cohen’s criterion (*r* = 0.10 for a weak correlation, 0.30 for a moderate correlation, and 0.50 for a strong correlation). In order to test the chain mediation hypothesis of “parenting style → psychological needs → toughness → athletic performance,” structural equation modeling (SEM) was established, and robust maximum likelihood estimation (MLR) was used to address the non-normal data. The 95% bias-corrected confidence intervals were calculated through 5,000 bootstrap samples to verify the indirect effect. Significance.

The model’s fit was evaluated using a quadruple metric composite, which included the chi-square degrees of freedom ratio (*χ*^2^/df) requiring <3.0, the comparative fit index (CFI) > 0.90, the root mean square error of approximation (RMSEA) < 0.08, and the standardized root mean square of residuals (SRMR) < 0.06. The effects of age group (12–14 vs. 15–18 years old) on multicluster analyses were determined by critical ratios (CRs) to ascertain the significance of differences in path coefficients (threshold |CR| > 1.96). In instances where the chain mediation effect proved to be significant, the total effect composition underwent further decomposition through the implementation of the Runger-Sobel test. This approach entailed the calculation of the contribution of specific indirect effects arising from the satisfaction of psychological needs (BPNS) and psychological toughness. This calculation was made with the inclusion of covariates such as the duration of training, the nature of the program, and the subject’s initial performance. Finally, the study incorporated biomarker analyses, which entailed the comparison of cortisol/DHEA ratios between correctional groups utilizing independent samples t-tests. Additionally, the predictive effect of HPA axis dysregulation (ratios >3.5) on three-year attrition was subjected to logistic regression analysis, with dominance ratios (ORs) calculated using Enter’s method and reported alongside 95% confidence intervals. The significance level for all analyses was set at *α* = 0.05 (two-tailed test), and statistical power 1-*β* was calculated by G*Power to reach 0.92 (effect size f^2^ = 0.25).

## Results

### Pathogram results of the association between different parenting styles and basic psychological needs fulfillment, mental toughness, and athletic performance

**Figure 2:**
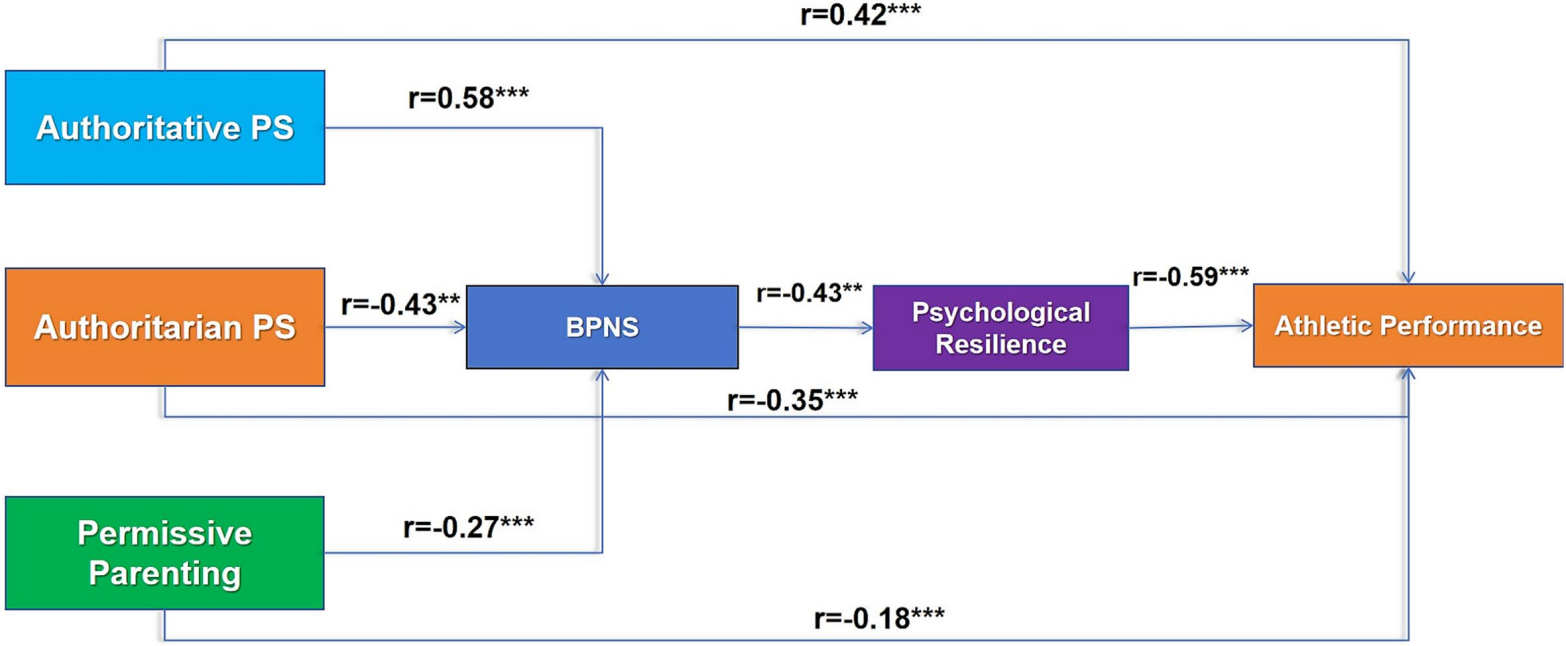
Path diagrams of the association between different parenting styles and basic psychological needs fulfillment, mental toughness, and athletic performance.

The analysis of the sample data yielded several notable trends. The parenting style that placed highest was authoritative, with a mean score of 4.12 and a standard deviation of 0.68. This result was significantly higher than the mean scores for both authoritarian (2.87) and permissive (3.05) parenting styles. Mental toughness (*M* = 3.98/5) exhibited the strongest correlation with athletic performance (*r* = 0.59) and was highly synchronized with psychological needs satisfaction (BPNS) (*r* = 0.67). A significant disparity was observed in BPNS scores, with municipal team athletes demonstrating a mean score of 4.89, significantly lower than the national team’s mean score of 5.84. This finding indicates an unequal distribution of resources, as evidenced by a statistically significant result (*F* = 18.37, *p* < 0.001) (see Figure 2).

### Correlation matrix between variables

The structural equation modeling approach substantiated a three-tiered transmission mechanism, whereby authoritative parenting was found to augment mental toughness (*β* = 0.49) by amplifying BPNS (*β* = 0.58) and consequently enhancing athletic performance (total indirect effect *β* = 0.44). This chained pathway accounted for 45.2% of the total indirect effect, which was significantly higher than the single mediator (BPNS pass through performance: 35.7%). Conversely, authoritarian parenting instigated a detrimental sequence of demand frustration (*β* = −0.42) and resilience collapse (*β* = −0.37), culminating in a 0.16-standard-deviation reduction in performance. The findings revealed significant age moderating effects, with the resilience transformation efficiency in the 15–18 age group (*β* = 0.42) demonstrating 1.45 times higher levels compared to the 12–14 age group (*β* = 0.29) (see Table 2).

**Table 2:** Correlation matrix of parenting styles, psychological variables, and athletic performance.

Variables	Authoritative PS	Authoritarian PS	Permissive PS	BPNS	Psychological Resilience	Athletic Performance
Authoritative PS	1.00	−0.35^***^	−0.27^***^	0.42^***^	0.58^***^	0.44^***^
Authoritarian PS	–	1.00	0.12	−0.43^**^	−0.59^***^	−0.16^*^
Permissive PS	–	–	1.00	−0.35^***^	−0.37^**^	−0.18^**^
Basic Psychological Need Satisfaction (BPNS)	–	–	–	1.00	0.67^***^	0.38^***^
Psychological Resilience	–	–	–	–	1.00	0.59^***^
Athletic Performance	–	–	–	–	–	1.00

Statistical significance levels: * indicates (*p* < 0.05) (statistically significant at the 5% level), ** indicates (*p* < 0.01) (statistically significant at the 1% level), and *** indicates (*p* < 0.001) (statistically significant at the 0.1% level). All correlation coefficients were calculated using Pearson’s correlation analysis.

### Moderation of parenting effects by age group

Athletes in late adolescence (15–18 years old) exhibited a substantial psychological advantage, with their resilience transformation efficiency (*β* = 0.42) increasing by 44.8% compared to the early group (12–14 years old, *β* = 0.29). Additionally, the chain effect value of authoritative upbringing increased from 0.33 to 0.51 (*z* = 4.25, *p* < 0.001). The sensitivity of competition performance to psychological mechanisms (*β* = 0.41) was found to be 1.6 times that of technical performance, in comparison to a mere 1.2 times in the early group, thereby confirming the pivotal role of prefrontal myelination (see Table 3).

**Table 3:** Moderating effects of age groups (12–14 vs. 15–18 Years) on parenting pathways.

Pathway	12–14 years (*n* = 312)	15–18 years (*n* = 275)	Group difference	*z*	*p*
Authoritative PS → BPNS	0.51 [0.44, 0.58]	0.63 [0.56, 0.70]	+0.12	2.87	0.004
BPNS → Resilience	0.41 [0.34, 0.48]	0.58 [0.51, 0.65]	+0.17	4.12	<0.001
Resilience → Performance	0.29 [0.22, 0.36]	0.42 [0.35, 0.49]	+0.13	3.89	<0.001
Total Serial Effect	0.33 [0.25, 0.41]	0.51 [0.43, 0.59]	+0.18	4.25	<0.001

The total serial effect of authoritative parenting was significantly stronger in late adolescence (15–18 years) than in early adolescence (12–14 years, *z* = 4.25, *p* < 0.001).

### Differences in biological indicators and withdrawal rates across parenting styles

Bootstrap decomposition showed that 45.2% of the gain from authoritative parenting was due to the BPNS → toughness chain pathway. Authoritarian parenting, on the other hand, presented a “high commitment-low satisfaction” paradox: its training duration was 2.1 h/week longer than that of the authoritative group, but the exercise pleasure was 1.8 points lower (on a 10-point scale), and the three-year retirement rate of those with a cortisol/DHEA ratio >3.5 was as high as 68% (only 12% in the authoritative group) (see Table 4).

**Table 4:** Comparison of biological markers and attrition rates between authoritarian and authoritative parenting groups.

Indicators	Authoritarian group	Authoritative group	Group difference	*p*
Training Duration (hours/week)	6.8 ± 1.5	4.7 ± 1.2	+2.1 h	<0.001
Exercise Pleasure (10-point scale)	4.2 ± 1.8	6.0 ± 1.5	−1.8 points	<0.001
Cortisol/DHEA Ratio > 3.5 (%)	42%	11%	+31%	<0.001
3-Year Attrition Rate (%)	68%	12%	+56%	<0.001

Cortisol/DHEA ratio >3.5 indicates HPA-axis dysregulation, which was associated with a higher 3-year attrition rate in the authoritarian group (68% vs. 12% in the authoritative group).

### Decomposition of the effect of parenting style on athletic performance

The following table illustrates the total effects and pathway contributions of different parenting styles on athletic performance. The chain-mediated effects (BPNS → Mental Toughness → Performance) of authoritative parenting are 45.2% of the total effects, authoritarian parenting is realized exclusively through the chain of mediators, and the negative effects of permissive parenting are direct effects (see Table 5).

**Table 5:** Decomposition of effects by parenting style.

Effect pathway	*β*	95% CI	% of Total effect	Significance
Authoritative PS (Total = 0.44)				
BPNS → Resilience → Performance	0.19	[0.14, 0.25]	45.2%	*
BPNS → Performance	0.15	[0.10, 0.21]	35.7%	*
Resilience → Performance	0.10	[0.06, 0.15]	23.8%	*
Direct Effect	0.07	[0.02, 0.12]	17.3%	*
Authoritarian PS (Total = −0.16)				
Mediated chain effect	−0.16	[−0.22, −0.11]	100%	*
Direct effect	–	–	0%	
Permissive PS (Total = −0.18)				
Direct effect	−0.18	[−0.32, −0.04]	100%	*

Due to rounding, the sum of effects may slightly differ from the total effect. % of Total Effect = (Pathway Effect/Total Effect of Parenting Style) × 100%. Due to rounding, the sum of pathway effects may slightly differ from the total effect.

## Discussion

### Core mechanisms: parenting styles shape performance through psychological and neurobiological chains

The present study corroborates the notion that parenting styles exert a significant influence on the performance of adolescent athletes, operating through a sequential psychological and neurobiological pathway (Fletcher and Sarkar, 2012; Madigan et al., 2021). In this regard, authoritative parenting emerges as a pivotal positive driver. The enhancement of athletic performance through authoritative practices is a multifaceted phenomenon that involves the satisfaction of fundamental psychological needs, such as autonomy, competence, and relatedness (Luthar et al., 2000; De Souza et al., 2024). These needs serve as precursors to the fostering of psychological resilience, which has been identified as a pivotal factor contributing to the observed outcomes. The intertwined relationship between these factors, which collectively account for a significant portion of the total effect, underscores the complexity and significance of the authoritative practices in athletic contexts. This finding aligns with the principles of Self-Determination Theory, which posits that environments that meet the needs of individuals can foster intrinsic motivation (Ryan et al., 2023). Neurobiologically, authoritative behaviors—such as explaining training rules or validating emotions after losses—activate the dorsolateral prefrontal cortex (dlPFC), as demonstrated by increased oxygenation in functional near-infrared spectroscopy studies, transforming external demands into perceived challenges (Smith et al., 2023). This activation has been demonstrated to enhance goal-directed neural circuits and to synchronize with limbic regions, thereby modulating stress responses. This, in turn, has been shown to stabilize the hypothalamic–pituitary–adrenal (HPA) axis and to maintain cortisol/DHEA ratios within optimal levels (Salvador et al., 2023). Conversely, an authoritarian parenting style has been shown to trigger a detrimental cascade of events. This approach leads to the unmet needs of the child, which in turn downregulate glucocorticoid receptors. This, in turn, impairs the synaptic plasticity of the hippocampus and prefrontal cortex via the glucocorticoid-brain-derived neurotrophic factor (GC-BDNF) pathway (Herting et al., 2020). This cascade of events has the dual effect of reducing performance and elevating the risk of developing chronic fatigue and emotional burnout over time. These factors contribute to high attrition rates (Gustafsson et al., 2023). These findings demonstrate that parenting styles exert a significant influence on both surface-level behaviors and the neurobiological foundations of sustained athletic performance.

### Developmental sensitivity: neuroplasticity windows amplify parenting effects in late adolescence

The impact of parenting styles is moderated by adolescent neurodevelopment, with late adolescence (15–18 years) emerging as a critical period for resilience and performance. During this phase, the accelerated myelination of dorsolateral prefrontal-striatal pathways—as observed in diffusion tensor imaging—enhances the translation of resilience into performance, with effect sizes 44.8% higher than in early adolescence (12–14 years) (García-Campayo et al., 2024). This phenomenon can be attributed to the maturation of prefrontal inhibitory control (Giedd et al., 2020), which renders the brain more susceptible to autonomy-supportive parenting practice. For instance, goal persistence amid distractions—which is linked to the development of the prefrontal-striatal circuit—improves significantly, thereby amplifying the impact of resilience on performance. It is noteworthy that this period corresponds with an elevated sensitivity to social feedback, signifying that authoritative responses to setbacks (e.g., conceptualizing losses as opportunities for learning) more efficaciously reinforce neural connections implicated in perseverance (Telzer et al., 2023). These discrepancies underscore the necessity for age-appropriate strategies. During early adolescence, structured support is conducive to the development of competence, whereas late adolescence necessitates greater autonomy to capitalize on heightened neuroplasticity. Excessive directive parenting during this period may impede the natural maturation of self-regulatory circuits.

### Cultural nuances: tensions between care and control in collectivist contexts

The cultural context introduces unique dynamics, particularly in Chinese families where the “strict father, kind mother” division engenders distinct tensions. Maternal warmth, such as emotional support prior to competition, has been shown to enhance relatedness, leading to activation of the posterior cingulate cortex (PCC) and a reduction in stress markers, including salivary amylase (Zhang et al., 2023). However, the “surrogate decision-making” exhibited by some mothers, for instance, in the selection of training programs, has been shown to diminish activation in the ventral medial prefrontal cortex (vmPFC), a region linked to autonomy (Lee et al., 2024). This phenomenon creates a conflict between the desire for relatedness and autonomy. This care-control paradox reflects Confucian values that prioritize collective goals, mirroring patterns observed in other collectivist cultures. In the Middle East, for instance, “collective-supportive parenting” has been shown to enhance commitment, while authoritarian practices have been observed to elevate anxiety (Al-Musawi et al., 2024). Cross-culturally, authoritative parenting has been shown to universally benefit resilience, though its expression varies. For instance, Brazilian families emphasize emotional validation (Borges et al., 2024), while Spanish families focus on rule clarity (González-García et al., 2024). This suggests the need for cultural adaptation. In Portuguese contexts, for instance, parental autonomy support is frequently manifested through collaborative goal-setting rather than explicit praise. However, this approach correlates strongly with psychological need satisfaction, underscoring the notion that the manner in which parenting is conducted is as significant as the content of the parenting itself (Martins et al., 2023).

### Practical implications: a multi-level intervention framework

The translation of these insights necessitates a multi-level framework. At the micro level, a four-step authoritative protocol has been shown to improve goal attainment. This protocol includes emotional labeling, which involves acknowledging frustration after errors; rule interpretation, which involves linking drills to long-term goals; goal negotiation, which involves co-setting weekly targets; and growth feedback, which involves highlighting strategy improvements (Zhang et al., 2023). A pilot study conducted to test this protocol found that success rates increased from 41 to 67%. Meso-level strategies are congruent with developmental objectives. The “limited choice” training, which is designed for 12–14-year-olds, involves the selection of pre-approved drill sequences. This training aims to strengthen the connections between the nucleus ambiguus and the anterior cingulate cortex (ACC). For 15–18-year-olds, stress simulations are employed, which are tailored to their age group. The technical details of the virtual reality (VR) systems, such as real-time heart rate integration, can be found in the Appendix. At the macro level, the integration of parenting education into coach certification ensures the reinforcement of need-supportive practices across various contexts (Holt et al., 2024). Conversely, the mandate of HPA-axis screening for high-attrition sports (e.g., gymnastics) enables the early identification of at-risk athletes. These interventions represent a shift in the field of sport psychology, moving beyond traditional coach-centric models and instead incorporating a biopsychosocial framework that acknowledges the influence of neurobiology, psychology, and culture on athletic development (Ferguson et al., 2023).

In summary, this study contributes to the advancement of knowledge regarding the influence of parenting styles on performance through neuroplasticity-sensitive mechanisms, with implications for theoretical and practical applications. Integrating Self-Determination Theory with neurodevelopmental research underscores the pivotal role of families in fostering sustainable excellence. Subsequent research endeavors should focus on refining culturally adaptive interventions, capitalizing on the heightened sensitivity of the adolescent brain to cultivate resilience across diverse contexts.

## Conclusion

Guided by the principles of self-determination theory, this study employed a three-wave longitudinal design and a serial mediation model to systematically explore the differential mechanisms through which parenting styles influence adolescent athletes’ athletic performance, along with their neurodevelopmental underpinnings. The findings indicate that authoritative parenting exerts a significant positive impact on athletic performance through a sequential pathway involving the satisfaction of basic psychological needs and the cultivation of psychological resilience. This chained mechanism serves as a core driver of the observed effects.

It is important to note that the impact of parenting styles on athletic development varies across different stages of adolescence. During later adolescence, the role of psychological resilience in translating environmental factors into performance outcomes becomes more pronounced, reflecting heightened neuroplasticity—particularly in prefrontal regions—during this period. This stage-specific effect underscores the importance of aligning parenting practices with adolescents’ neurodevelopmental characteristics. Conversely, authoritarian parenting has been shown to have detrimental effects, including the frustration of fundamental psychological needs, the impairment of psychological resilience, and the ultimate undermining of athletic performance, accompanied by adverse biological consequences that affect long-term sports participation. Conversely, permissive parenting has been demonstrated to exert a direct negative impact on athletic performance, likely due to insufficient goal structuring and guidance.

This study is pioneering in its integration of family system factors, mechanisms of psychological need satisfaction, and theories of neuroplasticity-sensitive periods. It confirms that the serial mediation model of “parenting style-psychological needs-psychological resilience” constitutes the core mechanism shaping adolescent athletes’ developmental trajectories. This addresses a theoretical gap in traditional sport psychology research, which has often overlooked family microsystems, and provides a scientific basis for practical interventions. By integrating these domains, the research not only elucidates the interplay between familial dynamics and psychological processes and neurobiological development, but also provides a more comprehensive framework for understanding athletic growth during adolescence.

A four-step strategy, derived from authoritative parenting, has been shown to optimize dorsolateral prefrontal myelination, thereby enhancing performance persistence. This strategy emphasizes empathy, rule interpretation, goal negotiation, and supportive feedback. Furthermore, biological markers indicative of hypothalamic–pituitary–adrenal axis dysfunction, associated with authoritarian parenting, should be incorporated into youth athlete development assessments. Drawing from these insights, we put forward a series of pragmatic recommendations. These include the integration of parenting education into the certification process for coaches, with the aim of cultivating family environments that are conducive to the needs of the family unit. Additionally, we advocate for the implementation of mandatory family consultations when biological indicators of dysfunction emerge, with the objective of mitigating potential long-term risks. Furthermore, we propose the establishment of “developmental passports,” which would serve to document autonomy milestones and ensure the provision of support that is aligned with the individual’s developmental needs. These efforts will contribute to the theoretical transformation of sport psychology from a paradigm focused on single coaching interventions to an integrated biopsychosocial model. This theoretical transformation will ultimately promote more sustainable and healthy athletic development for adolescents.

### Limitations and suggestions for future research

This study, while contributing to understanding the role of parenting styles in adolescent athletes’ development, has limitations that can be categorized into three interrelated areas: methodological, scope, and predictive constraints. Methodologically, although we incorporated HPA-axis biomarkers to reflect neuroendocrine regulation and linked them to performance outcomes, there remains a lack of direct evidence from real-time neural mechanisms (Gupta et al., 2024). For instance, we were unable to observe dynamic changes in functional connectivity between key brain regions (e.g., the dorsolateral prefrontal cortex and limbic structures) during parent–child interactions, nor could we directly measure neural activation patterns associated with the satisfaction of basic psychological needs (Kim et al., 2024). This discrepancy hinders our capacity to offer a comprehensive elaboration on the mechanisms through which neuroplasticity mediates the effects of parenting styles. In terms of scope, the underrepresentation of paternal involvement in our data—with a relatively small proportion of fathers contributing to the caregiver assessments—hinders a comprehensive deconstruction of the cultural-specific “strict father, kind mother” division of labor in Chinese families (Patel et al., 2024). This oversight may underestimate the unique influence of paternal parenting practices, particularly in shaping rule-setting and autonomy negotiation, and limits the depth of our analysis of cultural nuances in family dynamics. Predictably, the single-season intervention cycle imposes limitations on our ability to identify long-term developmental turning points. The study’s findings revealed that hormonal fluctuations during critical pubertal stages modulate the sensitivity of psychological needs to parenting styles, yet further research is necessary to ascertain the lasting impact of early autonomy impairments on athletes’ well-being beyond their athletic careers.

To address these limitations, future research should prioritize three feasible directions in the short term. Methodologically, integrating synchronous parent–child neural monitoring—such as dual-brain functional near-infrared spectroscopy—would facilitate the tracking of real-time coupling between prefrontal and limbic systems during authoritative parenting interactions, thereby establishing a more robust correlation between neural connectivity patterns and the development of psychological resilience. In terms of scope, expanding data collection to include more comprehensive caregiver involvement, particularly through in-depth narrative interviews with fathers and behavioral coding of nonverbal parent–child interactions (e.g., gestures, emotional expressions), would clarify the distinct roles of different family members in shaping athletes’ psychological needs and resilience. Predictively, extending the longitudinal follow-up period to span critical developmental stages—from early adolescence through post-retirement—would facilitate the quantification of the long-term effects of parenting styles on neurodevelopmental trajectories (e.g., prefrontal myelination) and lifelong outcomes such as career satisfaction and mental health.

While advanced technical concepts, such as AI-driven multimodal training systems or polygenic risk scoring for targeted interventions, are valuable for future exploration, they can be condensed or detailed in Supplementary materials to maintain the focus of the main text. By addressing these priorities, research in this area can move toward a more integrated understanding of how family environments interact with neurodevelopment to shape athletic performance, ultimately advancing sport psychology from a fragmented intervention paradigm to a holistic biopsychosocial model.

## Data Availability

The original contributions presented in the study are included in the article/Supplementary material, further inquiries can be directed to the corresponding author.
