# Supplementary material for: The mediating role of psychological resilience between parenting styles and athletic performance in adolescent athletes: a serial multiple mediation model
Source: Front Psychol. 2025 Sep 18;16:1661771. doi: 10.3389/fpsyg.2025.1661771 (PMC12490276; doi:10.3389/fpsyg.2025.1661771)
Supplement: Supplementary file 1 [file Supplementary_file_1.docx]

# APPENDIX S1: TECHNICAL SPECIFICATIONS OF INTERVENTION PROTOCOLS

**Four-Step Authoritative Parenting Protocol**

The comprehensive intervention protocol comprises four sequentially implemented components: emotional labeling, rule interpretation, goal negotiation, and growth feedback. Emotional labeling involves the identification and verbal articulation of negative emotions during training/competition, exemplified by parental responses such as 'I observe your frustration following that error' to athlete expressions like 'I feel disappointed after missing that shot'. Rule interpretation establishes explicit connections between immediate training tasks and long-term athletic development objectives, demonstrated through statements such as 'This passing drill enhances in-game offensive efficiency' from athletes and 'This week's endurance training directly prepares you for the final sprint in next month's championship' from parents. Goal negotiation employs SMART criteria (Specific, Measurable, Achievable, Relevant, Time-bound) for collaborative target-setting, where athlete-initiated goals ('I aim to increase serve accuracy from 70% to 80%') are met with parental scaffolding ('We agree on 20 additional daily serves with weekend progress assessments'). Growth feedback prioritizes process-oriented evaluation over outcome fixation using a progress-focused framework, with parental reinforcement ('Despite the loss, your defensive positioning showed clear improvement') following athlete self-reflection ('Adjusting my swing timing was strategically sound'). In the 8-week pilot implementation (March-May 2023) involving 72 gymnasts (M~age~=15.2±1.3 years), target attainment rates significantly increased from 41% to 67% (p<.001), with concomitant fNIRS-confirmed dlPFC oxygenation elevation of 18.3% (SD=3.1).

### APPENDIX S2: VIRTUAL REALITY STRESS SIMULATION SYSTEM ARCHITECTURE

**Technical Configuration and Operational Parameters**

The integrated VR platform utilizes HTC Vive Pro Eye HMDs (120° FoV, 90Hz refresh rate) synchronized with multi-modal biosensors: Polar H10 chest straps (heart rate acquisition at 130Hz sampling frequency) and Empatica E4 electrodermal activity monitors. The stressor repository incorporates three evidence-based competitive scenarios: 1) Referee error simulations combine erroneous score deductions with 85dB crowd disapproval to target amygdala-dlPFC emotional regulation circuits; 2) Critical point scenarios present tie-score conditions with coach countdown vocalizations ("Final shot!") to train attentional sustainability via parietal-anterior cingulate connectivity; 3) Injury return scenarios impose virtual mobility restrictions and 20% performance degradation to facilitate ventral striatal self-efficacy reactivation. The biofeedback algorithm initiates cognitive reappraisal training modules when real-time heart rate exceeds 160bpm, maintaining system-wide latency below 50ms through optimized GPU-accelerated rendering pipelines. Scenario validity was established through expert coach evaluations (ICC=.91) and cortisol-DHEA ratio confirmation (r=.73 with competition stress).

**HPA-axis Biomarker Assessment**

Salivary cortisol and DHEA were collected at 8 AM post-rest day using Salivette® tubes (Sarstedt), centrifuged (3,000 rpm × 15 min), and stored at -80°C. ELISA assays (DiaMetra kits DKO020/DKO034) had inter-assay CVs <8%. Cortisol/DHEA ratios >3.5 defined HPA-axis dysregulation.

### APPENDIX S3: ADVANCED TECHNICAL EXTENSIONS FOR FUTURE RESEARCH

**AI-Driven Multimodal Training Systems**

Future investigations should develop integrated artificial intelligence frameworks employing transformer-based neural architectures to synthesize multimodal athlete data streams. These systems would process synchronized neurophysiological inputs—including electroencephalographic (EEG) spectral features and 3D kinematic motion capture trajectories—alongside contextual behavioral markers such as coach feedback frequency and training diary annotations. Through hierarchical attention mechanisms, the models would generate personalized parenting strategy recommendations by identifying latent patterns between caregiver interaction styles and athlete neuro-cognitive profiles. Validation would require longitudinal testing across diverse sporting disciplines to establish predictive efficacy for resilience outcomes, with particular emphasis on detecting critical inflection points in prefrontal-striatal circuit development during high-stress competitive periods. Implementation pipelines must address ethical considerations regarding data privacy and algorithmic transparency through federated learning approaches and explainable AI visualization interfaces.

**Polygenic Risk Scoring Applications**

Precision intervention protocols could be enhanced through incorporation of polygenic risk scoring methodologies targeting neuroplasticity-associated genetic polymorphisms. Primary candidate variants include the BDNF-Val66Met (rs6265) single-nucleotide polymorphism influencing activity-dependent secretion of brain-derived neurotrophic factor and the COMT-Val158Met (rs4680) locus regulating prefrontal dopamine catabolism. Analysis workflows would commence with genome-wide association studies (GWAS) in athlete cohorts to identify sport-specific resilience polygenic architectures, followed by weighted polygenic score calculation using clumping and thresholding methods. Resultant risk stratification would enable matching of athletes to optimized parenting intervention modalities—for instance, carriers of high-risk BDNF Met alleles may derive greater benefit from autonomy-supportive scaffolding to counteract diminished activity-dependent neuroplasticity. Technical validation requires establishing measurement invariance across ethnic populations and controlling for epigenetic confounding through longitudinal methylome analysis. Implementation frameworks must integrate rigorous ethical oversight regarding genetic exceptionalism and biological determinism misconceptions.
